# Management guidelines for paediatric patients receiving chimeric antigen receptor T cell therapy

**DOI:** 10.1038/s41571-018-0075-2

**Published:** 2018-08-06

**Authors:** Kris M. Mahadeo, Sajad J. Khazal, Hisham Abdel-Azim, Julie C. Fitzgerald, Agne Taraseviciute, Catherine M. Bollard, Priti Tewari, Christine Duncan, Chani Traube, David McCall, Marie E. Steiner, Ira M. Cheifetz, Leslie E. Lehmann, Rodrigo Mejia, John M. Slopis, Rajinder Bajwa, Partow Kebriaei, Paul L. Martin, Jerelyn Moffet, Jennifer McArthur, Demetrios Petropoulos, Joan O’Hanlon Curry, Sarah Featherston, Jessica Foglesong, Basirat Shoberu, Alison Gulbis, Maria E. Mireles, Lisa Hafemeister, Cathy Nguyen, Neena Kapoor, Katayoun Rezvani, Sattva S. Neelapu, Elizabeth J. Shpall, the Pediatric Acute Lung Injury and Sepsis Investigators (PALISI) Network

**Affiliations:** 1 Department of Pediatrics, Stem Cell Transplantation and Cellular Therapy, CARTOX Program, The University of Texas MD Anderson Cancer Center, Houston, TX USA; 2 Department of Pediatrics, Blood and Marrow Transplantation Program, Keck School of Medicine, University of Southern California, Children’s Hospital Los Angeles, Los Angeles, CA USA; 3 Department of Anesthesiology and Critical Care, Division of Critical Care, University of Pennsylvania Perelman School of Medicine, Children’s Hospital of Philadelphia, Philadelphia, PA USA; 4 Department of Pediatrics, Division of Hematology–Oncology, University of Washington, Seattle Children’s Hospital, Seattle, WA USA; 5 Center for Cancer and Immunology Research and Department of Pediatrics, Children’s National and The George Washington University, Washington DC, USA; 6 Department of Pediatrics, Stem Cell Transplantation, Baylor College of Medicine, Texas Children’s Hospital, Houston, TX USA; 7 Pediatric Hematology–Oncology, Dana-Farber Cancer Institute, Harvard University, Boston, MA USA; 8 Department of Pediatric Critical Care, Weil Cornell Medical College, New York Presbyterian Hospital, New York, NY USA; 9 Department of Pediatrics, Division of Critical Care, University of Minnesota, Masonic Children’s Hospital, University of Minnesota, Minneapolis, MN USA; 10 Department of Pediatrics, Division of Critical Care, Duke Children’s Hospital, Duke University, Durham, NC USA; 11 Department of Pediatrics, Critical Care, CARTOX Program, The University of Texas MD Anderson Cancer Center, Houston, TX USA; 12 Department of Pediatrics, Neurology, CARTOX Program, The University of Texas MD Anderson Cancer Center, Houston, TX USA; 13 Department of Pediatrics, Division of Blood and Marrow Transplantation, Nationwide Children’s Hospital, the Ohio State University, Columbus, OH USA; 14 Department of Stem Cell Transplantation and Cellular Therapy, CARTOX Program, The University of Texas MD Anderson Cancer Center, Houston, TX USA; 15 Department of Pediatrics, Division of Blood and Marrow Transplant, Duke Children’s Hospital, Duke University, Durham, NC USA; 16 Department of Pediatrics, Division of Critical Care, St. Jude’s Children’s Research Hospital, Memphis, TN USA; 17 Department of Pharmacy, Children’s Hospital at Montefiore, Albert Einstein College of Medicine, Bronx, NY USA; 18 Department of Pharmacy, CARTOX Program, The University of Texas MD Anderson Cancer Center, Houston, TX USA; 19 Department of Lymphoma and Myeloma, CARTOX Program, The University of Texas MD Anderson Cancer Center, Houston, TX USA

**Keywords:** Haematological cancer, Cancer immunotherapy, Immunotherapy, Paediatric cancer, Adverse effects

## Abstract

In 2017, an autologous chimeric antigen receptor (CAR) T cell therapy indicated for children and young adults with relapsed and/or refractory CD19^+^ acute lymphoblastic leukaemia became the first gene therapy to be approved in the USA. This innovative form of cellular immunotherapy has been associated with remarkable response rates but is also associated with unique and often severe toxicities, which can lead to rapid cardiorespiratory and/or neurological deterioration. Multidisciplinary medical vigilance and the requisite health-care infrastructure are imperative to ensuring optimal patient outcomes, especially as these therapies transition from research protocols to standard care. Herein, authors representing the Pediatric Acute Lung Injury and Sepsis Investigators (PALISI) Network Hematopoietic Stem Cell Transplantation (HSCT) Subgroup and the MD Anderson Cancer Center CAR T Cell Therapy-Associated Toxicity (CARTOX) Program have collaborated to provide comprehensive consensus guidelines on the care of children receiving CAR T cell therapy.

## Abstract (web summary)

Chimeric antigen receptor (CAR) T cell therapies have impressive activity in the treatment of cancer but are associated with potentially fatal toxicities. In light of the approval of CAR T cell therapy for paediatric patients, a panel of experts from the Hematopoietic Stem Cell Transplantation (HSCT) Subgroup of the Pediatric Acute Lung Injury and Sepsis Investigators (PALISI) Network, the CAR T Cell Therapy-Associated Toxicity (CARTOX) Program at The University of Texas MD Anderson Cancer Center, and several other institutions have developed consensus guidelines for the use and management of these treatments in paediatric patients, which are presented herein.

## Introduction

Leukaemias are the most common childhood cancers, and acute lymphoblastic leukaemia (ALL) is the most common form of childhood leukaemia^1^. In the century following its first description, leukaemia remained a near uniformly fatal disease. In 1948, astute observations among children with leukaemia, made by Sidney Farber^2^, led to therapeutic breakthroughs — ushering in an era of hope. Since then, paediatric oncology cooperative group trials and pioneering work by E. Donnall Thomas on haematopoietic stem cell transplantation (HSCT) among patients with leukaemia have led to dramatic improvements in the cure rates of childhood ALL^3,4^. Despite improvements in overall remission rates (ORRs) for children with ALL, until now, the cure rates for children with relapsed and/or refractory disease remain disappointingly low^5,6^. In the 1970s, E. Donnall Thomas considered a 15% salvage rate after disease relapse to be a promising result among a patient population with previously even more dismal outcomes^7^. Since that time, durable remission rates have increased, with other dramatic improvements in supportive care, such as antimicrobial prophylaxis and therapy, infection control, HLA tissue typing, immunosuppression, transfusion support, and paediatric critical care management, resulting in improved patient survival.

In 2017, the FDA approved the first chimeric antigen receptor (CAR) T cell therapy, tisagenlecleucel, which has been associated with ORRs of almost 90% among children and young adults with B cell precursor ALL that is treatment refractory or in second or later relapse^8,9^. Notably, this novel genetically engineered, CD19-targeted, autologous T cell immunotherapy was approved specifically for a paediatric and young adult indication. While CAR T cell immunotherapy is an exciting paradigm shift in anticancer therapy, this treatment modality is associated with unique toxicities, which can lead to very rapid and life-threatening cardiorespiratory and/or neurological deterioration. Thus, this novel therapy requires the medical vigilance of a diverse multidisciplinary team — and the associated comprehensive clinical infrastructure — to ensure optimal outcomes.

CAR T cells are generated through genetic modification of the patient’s own (autologous) T cells or those of an allogeneic donor. The isolated cells are activated and genetically modified via viral transduction or nonviral gene transfer^10,11^. Specifically, the modified CAR T cells express an engineered chimeric cell-surface receptor comprising an extracellular antigen-recognition domain, which is usually an antibody single-chain variable fragment (scFv), linked to at least one intracellular signalling domain — usually the CD3ζ chain of the T cell receptor plus one or more domains derived from co-stimulatory receptors (such as CD28 or 4-1BB ligand receptor (4-1BB; also known as TNFRSF9)). The extracellular portion of the CAR enables recognition of a specific antigen (such as CD19), and the signalling domains stimulate T cell proliferation, cytolysis, and cytokine secretion to enable elimination of the target cell^12,13^. Autologous or allogeneic cells genetically engineered to express CARs targeting certain molecules commonly presented on the surface of cancer cells have been associated with durable remissions among patients for whom no alternative therapies were effective^14–27^.

Cytokine-release syndrome (CRS) and CAR T cell-related encephalopathy syndrome (CRES) are well-described unique toxicities associated with CAR T cells and some other immunotherapies^28–33^; however, the pathophysiological mechanisms of both CRS and CRES remain poorly understood. Results from animal studies reported in May 2018 implicate recipient monocyte-derived and/or macrophage-derived IL-1, IL-6, and nitric oxide (and not CAR T cell-derived cytokines) as the key determinants of the severity of CRS and CRES^34,35^. Indeed, CRS is a systemic inflammatory response caused by the CAR T cells and involving other immune cells that is typically characterized by fever, hypoxia, tachycardia, hypotension, and multi-organ dysfunction^36^. CRES can occur concurrently with CRS, following its resolution, or without associated CRS and is characterized by encephalopathy, delirium, seizures, and, rarely, cerebral oedema^37^. Almost half of all patients who receive tisagenlecleucel require intensive monitoring and critical care support, predominantly owing to these toxicities^9,38^. CRS and CRES are generally reversible but can be fatal. Paediatric-specific management guidelines, comprehensive training of interdisciplinary staff, effective communication, and an appropriately phased infrastructure to ensure that adequate resources are available should facilitate the early diagnosis and appropriate management of paediatric patients who develop CRS and/or CRES after receiving CAR T cell therapy as a standard of care or according to a research protocol — and thereby achieve optimal outcomes. Herein, we provide consensus guidelines for the use of CAR T cells in paediatric patients with cancer.

## Guideline formulation

A panel of experts from the HSCT Subgroup of the Pediatric Acute Lung Injury and Sepsis Investigators (PALISI) Network, the CAR T Cell Therapy-Associated Toxicity (CARTOX) Program at The University of Texas MD Anderson Cancer Center (Houston, TX, USA), and several other institutions with extensive interest and experience in CAR T cell therapy was convened to develop these guidelines. The PALISI Network includes clinical researchers from >90 paediatric intensive-care units across North America and beyond. The PALISI HSCT Subgroup is dedicated to improving the health and survival of children who require critical care during and following HSCT and cellular therapy through collaborative clinical and translational research. The guideline panel comprised a multidisciplinary and inter-professional team, including physicians with expertise in HSCT for paediatric and adult patients, translational immunotherapy researchers, paediatric intensivists, neurologists, paediatric nurses, advanced-practice providers, pharmacists, clinical nurse specialists and nursing administrators, and health-care administrators from a diverse group of medical centres. Using a modified Delphi method, the panel aimed to provide comprehensive guidelines for the safe administration of various CAR T cell therapies (standard of care and/or research protocol administration), the early recognition of CRS and/or CRES, and the management of these toxicities^39^. Levels of evidence and strength of recommendations were based on the classification schemes described by Shekelle and colleagues^40^ (Supplementary Table 1). A summary of our key recommendations and the associated level of supporting evidence are provided in Table 1.

**Table 1:** Summary of key recommendations for the use of CAR T cell therapy

Recommendations	Level of evidence	Grade
Providers are encouraged to adhere to product information labels and guidance from REMS programmes as they are approved by the FDA^8^	IV	D
Patient selection should be based upon the indications approved by the FDA and the criteria used in pivotal studies and can be tailored on the basis of emerging information from each new product^8,9,49^	IV	D
Consent should include descriptions of the risks and benefits associated with leukapheresis, lymphodepletion, CRS, CRES, bridging chemotherapy, intensive-care support (mechanical ventilation, dialysis, and inotropic support), and anti-IL-6 therapy^38^	IIA	B
When appropriate, child assent should also be obtained; age-appropriate advance directives should be considered. Incorporation of child life and psychological services in assent discussions can be helpful^44^	IV	D
Paediatric patients can require a leukapheresis catheter for cell collection. Close monitoring for hypotension, hypocalcaemia, and catheter-related pain is imperative during paediatric leukapheresis, particularly among infants and younger children who might not verbalize symptoms^47,48^	IIA	B
We recommend the selection of cyclophosphamide–fludarabine regimens for lymphodepletion, with exceptions considered in cases of haemorrhagic cystitis and/or resistance to a prior cyclophosphamide-based regimen^9,46,64,78–81^	IIA	B
Given the potential for rapid clinical deterioration, if CAR T cell therapy is administered in an outpatient setting, a low threshold should be set for patient admission upon the development of a fever and/or signs or symptoms that are suggestive of CRS and/or CRES^38^	IIA	B
On the basis of the published experience for tisagenlecleucel in paediatric and young adult patients with CD19^+^ relapsed and/or refractory B cell acute lymphoblastic leukaemia, considering inpatient admission for a minimum of 3–7 days following infusion is reasonable^8,38^	IIA	B
CRS grading should be performed as outlined in Table 2 at least once every 12 hours and more often if a change is noted and/or concerns exist^37^	IIA	B
Parent and/or caregiver concerns should be addressed because early signs or symptoms of CRS can be subtle and best recognized by those who know the child best^98^	III	C
CRS should be suspected if at least one of the following four symptoms or signs is present during the CRS-risk period within the first 2 weeks following CAR T cell infusion: fever ≥38 °C; hypotension (for patients aged 1–10 years: systolic blood pressure <(70 + (2 × age in years)) mmHg; for those aged >10 years: SBP <90 mmHg); a change from baseline and/or reduced requirements for chronic anti-hypertensive medications); hypoxia with an arterial oxygen saturation of <90% on room air; or evidence of organ toxicity as determined by the most recent CTCAE grading system (version 5.0)^99^ and paediatric considerations as outlined in Table 2 (refs^29,37,82^)	IIA	C
High vigilance for sinus tachycardia as an early sign of CRS is recommended (on the basis of age-specific normal range or baseline values)^103,104^	IIA	B
We recommend application of the PALICC at-risk P-ARDS criteria for the CRS grading of hypoxia^100–102^	IIA	B
Acute kidney injury in children can be graded according to CTCAE using pRIFLE and KDIGO definitions of oliguria^105,106^	IIA	B
Tocilizumab paediatric dosing: patients weighing <30 kg are dosed at 12 mg/kg, and those weighing ≥30 kg are dosed at 8 mg/kg (ref.^109^)	IIA	B
CAR T cell-related HLH and/or MAS have been shown to resolve following administration of anti-IL-6 therapy and corticosteroids, although refractory cases can require further therapy, including consideration of systemic and/or intrathecal therapy on the basis of HLH-2004 management guidelines or use of the IL-1 receptor antagonist anakinra; further research is needed in this area^62,113,114^	IIA	C
We recommend that delirium screening using the CAPD tool^116^ (or the CARTOX-10 grading system^37^ for patients aged ≥12 years who have sufficient cognitive abilities) be performed at least twice per 24-hour period among admitted patients and at least daily among outpatients during the high-risk periods for CRES	IIA	C
Consideration should be given to a prospective collaboration with intensive-care registries, such as VPS, which could allow accurate data entry of cell-therapy variables into the CIBMTR registry (by cell-therapy programmes) with concurrent entry of intensive-care variables into an appropriate registry by paediatric critical care teams	IV	D
We strongly encourage consideration of QALYs for paediatric patients who might achieve long-term remission through this therapy and encourage all efforts to reduce the cost of care^136–140^	IV	D
We recommend that CAR T cell programmes seek FACT IEC accreditation as a voluntary means of ensuring adherence to quality standards^55^	IV	D

Levels and grades of evidence have been assigned on the basis of the definitions proposed by Shekelle et al.^40^ (see Supplementary Table 1 for details). CAPD, Cornell Assessment of Pediatric Delirium; CAR, chimeric antigen receptor; CARTOX-10, CAR T Cell Therapy-Associated Toxicity 10-point assessment scale; CIBMTR, Center for International Blood and Marrow Transplant Research; CRES, CAR T cell-related encephalopathy syndrome; CRS, cytokine-release syndrome; CTCAE, Common Terminology Criteria for Adverse Events; FACT, Foundation for the Accreditation of Cellular Therapy; HLH, haemophagocytic lymphohistiocytosis; IEC, immune effector cell; KDIGO, Kidney Disease: Improving Global Outcomes; MAS, macrophage-activation syndrome; P-ARDS, paediatric acute respiratory distress syndrome; PALICC; Pediatric Acute Lung Injury Consensus Conference; pRIFLE, Pediatric Risk, Injury, Failure, Loss, End-Stage Renal Disease; QALYs, quality-adjusted life years; REMS, risk evaluation and mitigation strategy; SBP, systolic blood pressure; VPS, virtual paediatric intensive-care unit (PICU) Systems.

## Patient selection and evaluation

Currently, CAR T cell therapies are used mainly among patients with relapsed and/or refractory haematological cancers or other high-risk malignancies. As new genetically modified cellular therapies are developed, differences in a range of variables related to the source cell type (T cells, natural killer (NK) cells, natural killer T cells, and cytokine-induced killer cells, among others), the engineered product (for example, the co-stimulatory domains and gene transfer technologies used), the manufacturing process and reagents, the primary disease, and host factors might influence the individual toxicity profiles. Thus, providers of these treatments are encouraged to adhere to product information labels and guidance from risk evaluation and mitigation strategy (REMS) programmes as the products are approved by the FDA (Table 1).

Patient selection should be based upon the FDA-approved indications and eligibility criteria used in pivotal studies but could potentially be tailored on the basis of emerging information relating to each new product (such as label updates). Patients should generally have an acceptable performance status according to the thresholds defined in treatment protocols and/or institutional guidelines, which might vary for different indications and products and depending on whether any deficiencies are secondary to disease-specific manifestations and thus likely to improve with primary disease response. Patients should be evaluated for uncontrolled infections, active graft-versus-host disease (GVHD), or recent donor-lymphocyte infusion (DLI), with a threshold of at least 6 weeks between DLI and CAR T infusion^38^. Patients with uncontrolled infection and active grade II–IV acute or extensive chronic GVHD should be excluded. In patients with prior GVHD, the GVHD must have resolved and the patient should not have received systemic immunosuppression. Consideration should be given to the sites of active disease and specifically to whether immune activation, such as tumour flare ‘pseudoprogression’, could compromise vital organ function (for example, of the airway or central nervous system (CNS)). Patients with active CNS pathology should be selected with caution, particularly for products associated with CRES^41–43^. When feasible, baseline evaluation by interdisciplinary team members, such as intensive-care physicians and neurologists, could help to guide patient selection.

As new products are introduced, programmes should establish minimum eligibility criteria for patients to receive each agent. These criteria could potentially be adjusted over time on the basis of published experience gained in larger cohorts of patients. Patients identified as candidates for CAR T cell therapy should rapidly be referred for financial counselling to avoid delays in accessing care related to insurance pre-authorizations. For convenience, we have provided an overview of our general recommendation relating to CAR T cell-therapy eligibility and monitoring evaluations in the form of a checklist (Box 1).

Box 1 Paediatric patient selection, evaluation, and monitoring checklist for CAR T cell therapy **Patient selection and evaluation**

- Patients should have no evidence of uncontrolled infection or active graft-versus-host disease
- Patients should not have recently received therapy with donor-lymphocyte infusion
- Eligible patients who have previously received post-allogeneic haematopoietic stem cell transplantation immunosuppression should not be receiving immunosuppression before autologous leukapheresis
- For autologous chimeric antigen receptor (CAR) T cell production, an absolute lymphocyte count of >100 cells/µl can be acceptable (>500 cells/µl is generally preferred), but this varies depending on manufacturer guidelines
- Early referral for financial counselling
- Obtain consents (and child assent when appropriate)
- Infectious disease screening (for example, for hepatitis B virus (HBV) surface antigen, anti-HBV core antibodies, anti-hepatitis C virus (HCV) antibodies, and anti-HIV-1 and anti-HIV-2 antibodies, or using HIV, HBV, and HCV triple nucleic acid testing)

**Before lymphodepletion**

- Interval assessment with physical examination and screening for infection and organ toxicities
- Primary disease evaluation, for example, through lumbar puncture, bone marrow aspiration and biopsy, and PET–CT imaging
- Pregnancy test, if indicated, and confirmation of no substantial interval changes in height and/or weight
- Document baseline heart rate, blood pressure, mood, and cognitive and developmental status
- Establish central venous catheter or peripherally inserted central catheter access
- Confirm consents (and assents, if applicable) for the cell therapy and anti-IL-6 therapy (if applicable)
- Consider baseline assessment by neurologist and brain imaging (MRI or CT scan without contrast) and/or start levetiracetam for seizure prophylaxis^a^

**Before CAR T cells infusion**

- Confirm no uncontrolled infection; delay infusion if signs of uncontrolled infection are observed
- Consider raising an electronic medical record flag for CAR T cell therapy
- Obtain baseline vital signs
- Ensure that oxygen, suction pump, and emergency medications (such as adrenaline) are readily available
- At least two care providers with expertise in CAR T cell therapy should review the infusion order and CAR T cell product information
- Pre-medications should not include routine steroid administration
- Do not use a leukapheresis filter for infusion
- Be aware of management guidelines for infusion-related complications
- Infuse product according to manufacturer, protocol, and/or institutional guidelines
- Observe patient closely following infusion for infusion-related reactions
- Decide on inpatient versus outpatient monitoring on the basis of the toxicity profile of the specific CAR T cell product, the patient’s clinical status (assessed before and on the day of cell infusion) and psychosocial support network, and institutional outpatient infrastructure

**Post-infusion monitoring** ^***b***^

- Daily history and physical examination
- Daily complete blood count and blood product transfusion according to institutional guidelines for paediatric patients (without corticosteroid pre-medication)
- Daily monitoring for disseminated intravascular coagulation (prothrombin time, partial thromboplastic time, fibrinogen, and D-dimer testing)
- Daily monitoring for tumour-lysis syndrome (TLS) with basic metabolic panel, magnesium, phosphorus, uric acid, and lactate dehydrogenase measurements, and provide TLS prophylaxis if indicated
- Daily profiling of serum levels of liver enzymes, albumin, and fractionated bilirubin
- Daily serum C-reactive protein and ferritin monitoring for cytokine-release syndrome (CRS) and haemophagocytic lymphohistiocytosis
- Infectious disease (viral, bacterial, fungal, and parasitic) prophylaxis, including for *Pneumocystis jiroveci*, as appropriate
- Do not routinely administer corticosteroids (including as pre-medication)
- Perform CRS and CAR T cell-related encephalopathy syndrome (CRES) grading every 12 hours or more frequently with clinical status change (with outpatient management, consider including caregiver)
- Ensure anti-IL-6 therapy is available for ordering by cell-therapy physician
- Organ toxicity monitoring and grading according to the Common Terminology Criteria for Adverse Events version 5.0 (ref.^99^)
- Seizure prophylaxis with levetiracetam (10 mg/kg, up to a maximum of 500 mg per dose, every 12 hours for 30 days after CAR T cell infusion)^a^

**Admission orders (for patients who are admitted owing to toxicities including CRS and/or CRES)**

- Check vital signs every 4 hours (including pulse oximetry)
- Strongly consider continuous cardiopulmonary monitoring and consider telemetry (monitoring for hypoxia and dysrhythmias)
- Notify treating and/or attending physician of the following:

- Temperature >38 °C, and order blood cultures, urinalysis and urine culture, and chest radiography, and consider use of broad-spectrum antibiotics (especially for patients who are neutropenic)
- Heart rate or respiratory rate above or below age-specific normal range and/or baseline value (set range for sleeping and awake state)
- Systolic blood pressure (SBP) <(70 + (2 × age in years)) mmHg for patients aged 1–10 years; SBP <90 mmHg for those aged >10 years; for infants aged <1 year, SBP above or below age-specific normal range and/or baseline value
- Oxygen saturation <92% on room air
- Abnormal urine output according to age and weight (that is, none for 8 hours or <1 cc/kg per hour or >5 cc/kg per hour)
- CRS or CRES of any grade or any change in mental status (such as irritability or tremors)
- Upward trends in serum creatinine levels or detriments in liver function test results

^a^Recommended for patients treated with immune effector cell therapies known or suspected to cause CRES or first-in-human products and for patients with a predisposition to seizures. ^b^Post-infusion monitoring should continue until the patient has completed a high-risk CRS–CRES observation period (defined on the basis of data from the pivotal study of the agent of choice and emerging experience with similar products in similar patient populations).

## Informed consent and assent

CAR T cell therapy is a potentially curative treatment but is associated with life-threatening toxicities and can require long-term local follow-up evaluations and restrictions. Thus, detailed informed consent for therapy should be obtained from the patient and/or their guardians; when appropriate, child assent should also be obtained^44^ (Table 1). Incorporation of child life and psychological services in assent discussions might be helpful. In addition, capable patients should be asked to consider age-appropriate advanced medical directives. Even before leukapheresis is performed, the patient and/or their guardians should be informed about the potential benefits of CAR T cell therapy, as well as the potential toxicities and other risks associated with the procedure. Consent for CAR T cell manufacturing and therapy should encompass leukapheresis, lymphodepletion therapy, CRS and CRES, and the potential need for bridging chemotherapy, intensive-care support (including intubation and mechanical ventilation, vasopressor and/or inotropic support, renal replacement therapy, and intracranial hypertension management after transfer to the intensive-care unit), and anti-IL-6 therapy^38^. Patients should be aware that, even if the CAR T cell product is manufactured successfully, infusion of the product is contingent upon continued clinical eligibility. Patients should also be informed of the need to remain within 2 hours of the treatment facility for at least 4 weeks following infusion and of any other special precautions and limitations required during post-infusion monitoring after treatment with specific products (for example, limitation of driving)^8^.

## Leukapheresis for CAR T cell production

Generation of autologous CAR T cells requires the collection of CD3^+^ lymphocytes from the patient through leukapheresis. Absolute lymphocyte count (ALC) thresholds to proceed with leukapheresis can vary between different CAR T cell products; although an ALC of >100 cells/µl can be acceptable, >500 cells/µl (or an absolute CD3^+^ lymphocyte count of >150 cells/µl) is generally preferred^45,46^. Patients should undergo pre-collection testing to ensure that they are medically eligible to proceed with leukapheresis. Institutional or protocol-specific guidelines should outline haematological and other clinical criteria to proceed with collection. Paediatric patients can require a leukapheresis central venous catheter, rather than a peripheral venous cannula, for collection and should be haemodynamically stable (that is, able to tolerate fluid shifts) and free of uncontrolled infection^47–49^. The presence of infectious disease markers — for active or latent hepatitis B virus (HBV), active hepatitis C virus (HCV), or HIV — is generally assessed as part of the clearance protocol for leukapheresis. Close monitoring for hypotension, hypocalcaemia, and catheter-related pain is imperative during paediatric leukapheresis, particularly among infants and younger children who might not verbalize symptoms^50–54^ (Table 1). Packed red blood cells (irradiated) and/or albumin can be used to prime the collection in children weighing <30 kg. The targeted cell dose for leukapheresis can vary depending on the specific product and manufacturing process. Once an adequate quantity of cells has been collected, the cells are sent to the laboratory for CAR T cell manufacturing — a process that typically takes 2–4 weeks^8^. Cell-therapy production, storage, transportation, and shipping should occur in compliance with the most current standards as defined by the Foundation of Accreditation of Cellular Therapy (FACT)^55^.

## Allogeneic CAR T cells

Limitations of autologous CAR T cell therapy include the time required after leukapheresis to manufacture the cell product (especially among patients with advanced-stage disease and a very narrow therapeutic window), the cost of manufacturing a patient-specific product, and the fact that adequate leukapheresis might not always be possible among heavily pretreated patients. Allogeneic CAR cells would presumably offer an ‘off-the-shelf’, third-party approach to CAR-based cell therapy^56–59^. CAR-transduced cord blood-derived NK cells have been reported to have potent anticancer effects in preclinical studies^60^. This allogeneic NK cell approach to CAR cell therapy might also be associated with a reduced risk of GVHD compared with the use of allogeneic T cells. Moreover, the NK cell product used included a ‘suicide’ gene — inducible caspase 9 — that could be pharmacologically activated to eliminate the transduced cells, thus providing an additional safety mechanism^60^. A clinical trial to test the safety and efficacy of this product is currently underway at the MD Anderson Cancer Center (NCT03056339). In other allogeneic CAR-expressing cell-therapy approaches, functional co-expression of RQR8, a construct combining epitopes from CD34 and CD20, renders the CAR cells sensitive to the monoclonal anti-CD20 antibody rituximab, as a safety feature^56,61^. Safety mechanisms designed to eliminate transduced cells have also been added to autologous CAR-expressing cells, such as anti-CD19 CAR T cells also expressing inactive, truncated EGFR, which enables the cells to be targeted through administration of the anti-EGFR antibody cetuximab^49^. The recommendations provided herein should be applicable to both autologous and allogeneic CAR-based cell therapies.

## Bridging chemotherapy

Some patients with high-risk advanced-stage malignancies require bridging chemotherapy in the period immediately following leukapheresis. The goal of bridging chemotherapy is to maintain disease control and prevent progression (in addition to potentially decreasing tumour burden, which might reduce the risk of severe CRS^20,29,62^) while the autologous CAR T cells are manufactured rather than to act as a primary treatment of the disease^20,62^. During this 2–4-week period, patients should be monitored for tumour-lysis syndrome (TLS) and should receive antimicrobial prophylaxis with routine infection precautions^63^. Indeed, the bridging chemotherapy regimen should be selected carefully during this critical period in order to minimize the risk of toxicities, which might disqualify the patient from proceeding to lymphodepletion and/or CAR T cell infusion^45^. Several bridging chemotherapy regimens are commonly used in paediatric patients (Box 2); however, insufficient data are currently available to recommend an optimal regimen. This choice might be influenced by certain study protocol guidelines, characteristics of the particular CAR T cell product, and/or patient-specific clinical variables (such as prior response to particular chemotherapeutic agents and baseline organ function).

Box 2 Common bridging chemotherapy regimens for paediatric patients **Systemic chemotherapy**

- Cytarabine 300 mg/m^2^ and etoposide 150 mg/m^2^ intravenous (i.v.) daily for 3–5 days, with or without a single dose of polyethylene glycol (PEG)ylated asparaginase 2,500 units/m^2^ intramuscular (i.m.) 24–48 hours after cytarabine (or Erwinia asparaginase 25,000 units/m^2^ i.m. if patient is allergic to PEG)
- Vincristine 1.5 mg/m^2^ (maximum dose 2 mg) i.v. weekly for 4 doses and dexamethasone 6 mg/m^2^ (i.v. or orally) daily for 5 days
- Attenuated VAD: vincristine 1.5 mg/m^2^ (maximum dose 2.0 mg) i.v. weekly for 4 doses, dexamethasone 6 mg/m^2^ (i.v. or orally) daily for 5 days, and doxorubicin 50 mg/m^2^ i.v. (single dose in first week only)
- Continuous daily 6-mercaptopurine at 50 mg/m^2^ orally
- Continuous daily hydroxyurea (can titrate dose between 15 and 50 mg/kg per day orally)
- Attenuated FLAG: fludarabine 25 mg/m^2^ i.v. daily and cytarabine 2 g/m^2^ i.v. daily, both for 2–5 days, followed by filgrastim 5 µg/kg daily until absolute neutrophil counts reach >1,000 cells/µl for 2 consecutive days or until the day before the start of lymphodepletion
- Cyclophosphamide 1,000 mg/m^2^ i.v. (single dose) and cytarabine 75 mg/m^2^ i.v. daily for 4 days; may add 6-mercaptopurine 60 mg/m^2^ daily for 14 days at treating physician’s discretion
- Tyrosine kinase inhibitors (TKIs), either as monotherapy or in combination with chemotherapy, can be considered for patients with Philadelphia chromosome (Ph)-positive or Ph-like acute lymphoblastic leukaemia
**Intrathecal chemotherapy (intrathecal methotrexate monotherapy or intrathecal triple therapy)**
- Age 0–0.99 years: methotrexate 7.5 mg with or without hydrocortisone 7.5 mg and/or cytarabine 15 mg
- Age 1–1.99 years: methotrexate 8 mg with or without hydrocortisone 8 mg and/or cytarabine 16 mg
- Age 2–2.99 years: methotrexate 10 mg with or without hydrocortisone 10 mg and/or cytarabine 20 mg
- Age 3–8.99 years: methotrexate 12 mg with or without hydrocortisone 12 mg and/or cytarabine 24 mg
- Age ≥9 years: methotrexate 15 mg with or without hydrocortisone 15 mg and/or cytarabine 30 mg
**General recommendations regarding the timing of treatment discontinuation**
- TKIs and hydroxyurea must be stopped ≥72 hours before chimeric antigen receptor (CAR) T cell infusion
- The following drugs must be stopped ≥1 week before CAR T cell infusion: vincristine, 6-mercaptopurine, 6-thioguanine, methotrexate ≤25 mg/m^2^, cytarabine ≤100 mg/m^2^, and asparaginase (non-PEGylated)
- The following drugs must be stopped ≥2 weeks before CAR T cell infusion: clofarabine, cytarabine >100 mg/m^2^, anthracyclines, cyclophosphamide, and methotrexate ≥25 mg/m^2^
- PEGylated asparaginase must be stopped ≥4 weeks before CAR T cell infusion
- Central nervous system prophylaxis treatment must be stopped ≥1 week before CAR T cell infusion

## Preparative lymphodepletion treatment

Several lymphodepletion regimens are commonly used in the treatment of paediatric patients^9,22,64^ (Box 3). The T cell pool is subject to homeostatic regulation; therefore, a lymphopenic environment might be favourable for adoptive T cell transfer owing to reduced competition for homeostatic factors. Indeed, the removal of ‘cytokine sinks’ increases the availability of cytokines that promote lymphocyte proliferation and survival, such as IL-7 and IL-15, and can be a contributing factor to the effectiveness of tumour-specific T cells^65–67^. Furthermore, the depletion of immunosuppressive CD4^+^CD25^+^ regulatory T cells^68–75^ has been proposed as a key mechanism by which lymphodepletion augments adoptive T cell therapy^76,77^. Accordingly, lymphodepletion has also been shown to improve the expansion and persistence of adoptive CAR T cells and to enhance their anticancer efficacy, resulting in increased ORRs^64,66^. Of note, the majority of paediatric patients enrolled in the trials of tisagenlecleucel completed lymphodepletion before CAR T cell infusion^9,38^. We recommend that all patients undergo lymphodepletion if possible (omission of lymphodepletion might need to be considered in patients with lymphopenia).

Cyclophosphamide-based lymphodepletion regimens are commonly used before CAR T cell infusion, and the addition of fludarabine to lymphodepletion chemotherapy has been associated with improved CAR T cell expansion and persistence and prolongation of disease-free survival in patients with ALL^64,78^. Alternative lymphodepletion regimens (Box 3) can be considered in patients with haemorrhagic cystitis and/or resistance to a prior cyclophosphamide-based regimen^9,46,64,79–81^ (Table 1).

To proceed with lymphodepletion and CAR T cell infusion, patients should not have uncontrolled infection because hyperinflammatory states pre-infusion have been associated with increased risks of morbidity and mortality^49,82–84^. Thus, an interval assessment should be performed on the day of initiation of the lymphodepletion regimen to identify any new complications. This evaluation usually includes screening for signs and symptoms of active infection and/or new organ toxicity, as well as exclusion of pregnancy if indicated (Box 1). Anticipatory guidance regarding the adverse effects of the specific drugs and treatment modalities used should be reviewed. Haemodynamic and laboratory monitoring and hydration should be tailored on the basis of the selected lymphodepletion regimen.

Box 3 Lymphodepletion chemotherapy regimens used in paediatric patients **Cyclophosphamide plus fludarabine**

- Cyclophosphamide 900 mg/m^2^ (single dose) and fludarabine 25 mg/m^2^ daily for 3 days^22^
- Cyclophosphamide 30–60 mg/kg (single dose) and fludarabine 25 mg/m^2^ daily for 3 days^64^
- Cyclophosphamide 30–60 mg/kg (single dose) and fludarabine 25 mg/m^2^ daily for 5 days^64^
- Cyclophosphamide 500 mg/m^2^ daily for 2 days and fludarabine 30 mg/m^2^ daily for 4 days^9^
- Cyclophosphamide 300 mg/m^2^ daily for 3 days and fludarabine 30 mg/m^2^ daily for 3 days^9^
**Cyclophosphamide plus etoposide**
- Cyclophosphamide 440 mg/m^2^ daily for 2 days and etoposide 100 mg/m^2^ daily for 2 days^9^
- Cyclophosphamide 2–4 g/m^2^ (single dose) and etoposide 100 mg/m^2^ daily for 3 days^64^
**CVAD A**
- Cyclophosphamide 300 mg/m^2^ every 12 hours on days 1–3, vincristine 1.5 mg/m^2^ (maximum 2 mg) on day 3, and adriamycin 50 mg/m^2^ on day 3 (REF.^9^)
**CVAD B**
- Methotrexate 1 g/m^2^ on day 1 and cytarabine 1 g/m^2^ every 12 hours on days 2 and 3 (ref.^9^)
**Cytarabine plus etoposide**
- Cytarabine 300 mg/m^2^ (single dose) and etoposide 150 mg/m^2^ (single dose)^9^
**Cyclophosphamide monotherapy**
- 2–4 g/m^2^ (single dose)^64^
- 1 g/m^2^ (single dose)^9^
- 300 mg/m^2^ every 12 hours for 3 days^9^

**Clofarabine**

30 mg/m^2^ daily for 5 days^9^

## Cell infusion

Patients should not have evidence of uncontrolled infection and/or other contraindications before CAR T cell infusion. Contraindications include active or latent HBV infection, active HCV or HIV infection, severe acute or chronic extensive GVHD, and pregnancy^8,37,38,49,82–85^. If any of these features are present, CAR T cell infusion should be delayed to avoid potentially severe immune activation and associated sequelae.

At the time of infusion, oxygen, suction, and emergency medications, including adrenaline, should be readily available (Box 1). The patient and/or their caregiver should be instructed to report symptoms, such as shortness of breath, rash, chills, chest pain, and back pain. Infusion should occur through the largest patent lumen without a filter and generally without an infusion pump. Pre-medication with drugs including acetaminophen and diphenhydramine should be administered 30–60 mins before CAR T cell infusion^8^ in order to prevent infusion reactions related to cryopreservants, such as dimethyl sulfoxide. Corticosteroids should not be routinely used for pre-medication, as these agents are lymphocytotoxic and, thus, their administration early in the treatment course (before CAR T cell expansion in vivo) could affect therapeutic outcomes^20^. With the use of CAR T cell products approved by the FDA, we recommend following the FDA-approved package labelling; otherwise, protocol-specific guidelines should be followed. Vital signs and urine output should be monitored closely after the time of infusion.

Infusion of cellular therapy products is generally safe, although serious adverse infusion reactions can occur. Characteristic adverse reactions include nausea, vomiting, abdominal pain, chills, fever, and, rarely, severe respiratory depression, neurotoxicity, and cardiac arrhythmias^86–93^. General management principles for infusion reactions associated with adoptive cell therapy include consideration of slowing or halting the infusion, activation of emergency precautions, and confirmation of product details for accuracy. If symptoms resolve uneventfully without medical intervention, a serious infusion-related event is unlikely to have occurred. If an infusion-related event is considered likely, a transfusion-reaction laboratory evaluation and appropriate supportive care according to institutional guidelines should be initiated^94^. Bacterial infusion reactions occur from infusion of contaminated products, typically with Gram-negative organisms; therefore, prompt treatment with appropriate antibiotics and supportive care for fever, severe hypotension, and other unexpected signs and symptoms is important to preclude clinical deterioration and potentially death.

## Inpatient and outpatient management

Early recognition of toxicities of CAR T cell therapy, particularly CRS and/or CRES, in paediatric patients requires detection of variations from baseline in characteristics including heart rate, blood pressure, temperature, and irritability (Box 1). Both the efficacy and toxicity profiles of CAR T cell therapy might vary depending on the specific product administered and individual patient characteristics. Thus, the decision on inpatient versus outpatient management of patients treated with CAR T cell therapy should involve consideration of the toxicity profile of the product used, the clinical status of the patient (including risk of TLS), and the ability of the institution to deliver prompt and comprehensive outpatient management, as well as the ability of the patient to access such care. Given the potential for rapid clinical deterioration, if CAR T cell therapy is administered in an outpatient setting, a low threshold should be used for patient admission upon development of fever and/or other signs or symptoms that are suggestive of CRS and/or CRES^38^ (Table 1). The presence of a reliable, consistent, and well-informed caregiver is essential to facilitate outpatient administration of CAR T cell therapy. In instances in which patients are not admitted for CAR T cell infusion and CRS–CRES monitoring, adequate outpatient facilities are needed, with extended outpatient hours (as defined by the institutions), prompt access to emergency and critical care, and trained staff who are knowledgeable of CAR T cell toxicity and are capable of prompt patient evaluation and management available at all times. This requires adequate outpatient space with a design appropriate to the protection of patients who are immunocompromised while they are being triaged. Moreover, rapid access to a cellular therapy physician, and pharmacy, laboratory, and transfusion medicine services should be guaranteed.

As products transition from research study protocols to standard-of-care administration, institutional guidelines should consider whether most patients require inpatient hospitalization during the pivotal trials and/or what infrastructure is needed for outpatient administration. For example, during the international phase II ELIANA study of tisagenlecleucel in paediatric and young adult patients with CD19^+^ relapsed and/or refractory B cell ALL^38^, 76% of patients underwent cell infusions in the inpatient setting. CRS occurred in 77% of patients, with a median time to onset of 3 days (range 1–22 days)^38^. Almost half of all patients required intensive-care support, with a median stay of 7 days (range 1–34 days); intensive-care support included the use of high-dose vasopressors, oxygen supplementation, mechanical ventilation, and/or dialysis^38^. Neurological events occurred in 40% of patients, with the majority of cases occurring concurrently with, or soon after resolution of, CRS^38^. Two deaths occurred within 30 days of CAR T cell infusion (one patient died of cerebral haemorrhage and another died of progressive leukaemia)^38^. On the basis of the published experience with this product, considering inpatient admission for a minimum of 3–7 days following infusion is reasonable, especially as this treatment is increasingly being offered as a standard of care^8,38^ (Table 1). Nevertheless, the length of inpatient hospitalization and/or need for daily outpatient assessments might vary on the basis of the risks of CRS and CRES, the clinical and developmental status of the patient, and the social support systems available to the patient at home. The risk of developing CRS and/or CRES probably depends on the patient, the source of immune effector used to manufacture the CAR T cell therapy, the specific CAR T cell product, and/or the associated lymphodepletion strategy.

Admission orders and handoff communication should include information regarding the patient’s baseline heart rate, blood pressure, and mood, cognition, and developmental status. In addition to the aforementioned screening (Box 1), continuous cardiac — and, if feasible, telemetry — monitoring should be strongly considered, beginning on the day of CAR T cell infusion (to ascertain baseline physiology) and continuing for a minimum number of days following infusion (based upon peak incidence of CRS and/or CRES) or until any emergent CRS resolves. Sinus tachycardia can be an early presenting sign of CRS^29,95^, and recognition of this clinical finding requires high vigilance and awareness of the child’s baseline heart rates as well as age-specific normal values. To enable prompt medical intervention for these toxicities, we recommend that patients have central venous access or a double or triple lumen peripherally inserted central catheter^37^. For patients with a history of, or predisposition to, seizures or those with a high risk of CNS pseudoprogression (such as those with CNS disease, chloromas and/or leptomeningeal enhancements, or a prior history of seizures), a baseline neurology evaluation, electroencephalography (EEG), and/or baseline MRI of the brain and spinal cord and/or anti-seizure prophylaxis should be considered^96^.

We recommend that all patients have frequent physical examinations and laboratory monitoring with complete blood counts, comprehensive metabolic panels, coagulation testing, and serum ferritin and C-reactive protein measurements during the post-infusion period associated with a high risk of CRS (as defined in pivotal studies and by emerging data for each product)^37,82,84^ (Box 1). Regular — daily, if possible — monitoring for TLS is also important, and TLS prophylaxis is recommended for patients with a high disease burden^16,29,37,82,84,96^. In addition, infectious disease prophylaxis against viral, bacterial, and/or fungal pathogens should be prescribed as appropriate^63,82^. Patients should have adequate hydration with monitoring for acute fluid overload (daily weights and fluid intake–output recording). If needed, transfusions (irradiated blood products) should be ordered according to institutional guidelines for paediatric patients (without routine corticosteroid pre-medication). The risk of bleeding can be exacerbated by hypofibrinogenaemia and/or thrombocytopenia, especially in patients receiving anti-coagulation therapy (including through continuous venovenous haemofiltration)^38,82,97^. Conservative management of bleeding or hypofibrinogenaemia and/or thrombocytopenia, with the use of cryoprecipitate or fresh frozen plasma, as needed, is recommended to avoid lethal haemorrhage^82^. Administration of growth factors (G-CSF) should be considered for patients with neutropenic fever^8,82^. Conditional orders for fever and/or neutropenia and suspected sepsis can enable rapid intervention, when needed, by the nursing unit, outpatient triage personnel, and pharmacy. We recommend creation of a flag or banner in the electronic health system to alert care providers of patients who are CAR T cell recipients. Indeed, recipients of CAR T cell products should be made easily identifiable; in particular, the strong contraindication of these patients to steroids must be highly visible to avoid routine or accidental administration of these drugs (for example, during blood product and other pre-medication orders). An ‘as needed’ order, which requires a real-time electronic approval by an authorized care-provider, is recommended in order to ensure rapid access to the correct dose of anti-IL-6 therapy (for example, with the anti-IL-6 receptor antibody tocilizumab) when required for the treatment of CRS and/or CRES.

## CRS monitoring, grading, and management

CRS reflects a systemic inflammatory response driven by rapid and excessive secretion of cytokines (a so-called cytokine storm) that is associated with a spectrum of symptoms ranging from fever to multi-organ dysfunction^34,35^. As mentioned previously, 77% of the paediatric and young adult population of the ELIANA trial developed CRS after treatment with tisagenlecleucel, with almost half experiencing severe symptoms requiring intensive-care support^38^. In addition, 40% of patients developed CRES (grade 3 in 13%)^38^. Other notable adverse events included infection (in 43% of patients), cytopenias (grade ≥3 neutropenia and thrombocytopenia not resolved by day 28 in 35% and 7%, respectively), and TLS (in 4%)^38^. Patients with a high risk of developing severe CRS include those with early symptom onset (typically within 3 days of CAR T cell infusion), a high disease burden, and/or pre-existing comorbidities^9,20,22^.

Early detection of CRS and/or CRES in paediatric patients can be challenging; however, early diagnosis of CRS and its prompt management can mitigate the risks of life-threatening sequelae. We recommend that patients who show signs of CRS and/or CRES be admitted for observation. The grading and management of CRS have been largely based on criteria originally outlined by Lee and colleagues^29^. In the January 2018 issue of this journal, Neelapu and other members of the MD Anderson Cancer Center CARTOX Program published updated and more-detailed guidelines for the management of adult patients with CRS^37^. Herein, these recommendations for adult patients have been modified with input from the PALISI Network HSCT Subgroup to provide paediatric-specific guidelines.

CRS grading according to the criteria outlined in Table 2 should be performed at least once every 12 hours and more often if a change in the patient’s clinical status or reasons for concern are noted (Table 1). Parent and/or caregiver concerns should be thoroughly investigated because early signs or symptoms of CRS can be subtle and thus might be best recognized by those who know the child very well^98^ (Table 1). For example, detection of CRS involving the gastrointestinal system often requires recognition of changes in the child’s food intake and/or the frequency and consistency of bowel movements, as well as expression of nausea. We recommend that CRS grading performed primarily by physicians, advanced-practice providers, and bedside nurses be reviewed by interdisciplinary team members immediately after each assessment and, when possible, should include participation of the patient and/or parent or caregiver at the bedside. Nurses should ideally perform assessments mid-shift and jointly with incoming nurses during handoff at the end of their shifts. In the outpatient setting, properly trained caregivers could potentially perform CRS–CRES assessment in lieu of twice-daily clinical assessments by a health-care professional, but this approach has not been validated.

**Table 2:** Cytokine-release syndrome grading and management

Grade 1 CRS	Grade 2 CRS	Grade 3 CRS	Grade 4 CRS
Signs and symptoms			
• Temperature ≥38 °C • No hypotension • No hypoxia • Grade ≤1 organ toxicity^a^	Any temperature and any of the following: • Hypotension that responds to i.v. fluids or low-dose vasopressor treatment • SpO_2_ <90% on room air: FiO_2_ requirement <40% to keep SpO_2_ >88% • Grade 2 organ toxicity^a^	Any temperature and any of the following: • Hypotension (age 1–10 years: SBP <(70 + (2 × age in years)) mmHg; age >10 years: SBP <90 mmHg) requiring high-dose or multiple vasopressors • FiO_2_ requirement ≥40% and/or requiring BiPAP to keep SpO_2_ >88% • Grade 3 organ toxicity^a^ • Grade 4 transaminitis (>20× ULN)	Any temperature and any of the following: • Persistent hypotension despite fluid resuscitation and treatment with multiple vasopressors • Requirement for invasive mechanical ventilation • Grade 4 organ toxicity^a^ (except grade 4 transaminitis)
Paediatric considerations			
• Asymptomatic sinus tachycardia is defined by heart rates above the age-specific normal range or baseline values)	• Hypotension is defined as follows: SBP <(70 + (2 × age in years)) mmHg in patients aged 1–10 years; SBP <90 mmHg in patients aged >10 years	• Oliguria is defined as a urine output of <0.5 ml/kg per hour for 8 hours	• Anuria is defined as a urine output of <0.3 ml/kg per hour for 24 hours or 0 ml/kg per hour for 12 hours
Management			
• Acetaminophen, as needed, for fever • Evaluate for infectious aetiologies (blood and urine cultures and chest radiography) • Consider broad-spectrum antibiotics and filgrastim (if patient is neutropenic) • Assess for adequate hydration • Consider anti-IL-6 therapy for persistent or refractory fever^b^ • Symptomatic management of constitutional symptoms and organ toxicities	• Manage according to recommendations for grade 1 CRS (if applicable) • Administer i.v. fluid bolus of 10–20 ml/kg normal saline; repeat as necessary to maintain SBP above baseline or age-specific normal range • For hypotension refractory to fluid boluses or hypoxia, consider anti-IL-6 therapy with i.v. tocilizumab (12 mg/kg for patients weighing <30 kg or 8 mg/kg for those weighing ≥30 kg, to a maximum of 800 mg per dose); repeat dose every 8 hours for up to 3 doses within 24 hours (but titrate frequency according to response) • If hypotension persists after two fluid boluses and anti-IL-6 therapy, start vasopressors, transfer patient to PICU, and obtain echocardiogram • Use supplemental oxygen as needed • If patient is at high risk of severe CRS^c^, hypotension persists after anti-IL-6 therapy, or there are signs of hypoperfusion or rapid deterioration, use stress-dose hydrocortisone (12.5–25 mg/m^2^ per day divided every 6 hours; i.v. dexamethasone 0.5 mg/kg (maximum 10 mg per dose) every 6 hours; or methylprednisolone 1–2 mg/kg per day divided every 6–12 hours)	• Manage according to recommendations for grades 1 and 2 CRS • Transfer patient to PICU and obtain echocardiogram, if not performed already • Administer i.v. dexamethasone 0.5 mg/kg (maximum 10 mg per dose) every 6 hours; can increase dose to maximum of 20 mg every 6 hours if patient is refractory to lower dose (alternatively, methylprednisolone 1–2 mg/kg per day divided every 6–12 hours can be used)^d^ • Use supplemental oxygen, including high-flow oxygen delivery and non-invasive positive pressure ventilation	• Administer i.v. fluids, anti-IL-6 therapy, corticosteroids, and vasopressors and perform haemodynamic monitoring as described for grades 1, 2, or 3 CRS • If low doses of corticosteroids do not lead to clinical improvement, consider high-dose methylprednisolone (1 g daily for 3 days followed by rapid taper on the basis of clinical response)

Early recognition of cytokine-release syndrome (CRS) and appropriate intervention are essential to avoid life-threatening complications of this toxicity. CRS should be suspected if any of the above listed signs and symptoms are present within the first 3 weeks after chimeric antigen receptor (CAR) T cell therapy. CRS grading should be performed at least twice a day and when a change in the patient's clinical status occurs. BiPAP, bi-level positive airway pressure; FiO_2_, fraction of inspired oxygen; i.v., intravenous; PICU, paediatric intensive-care unit; SBP, systolic blood pressure; SpO_2_, peripheral capillary oxygen saturation; ULN, upper limit of normal.

^a^Graded according to the Common Terminology Criteria for Adverse Events version 5.0 guidelines^99^.

^b^For example, persistent fever lasting >3 days or fever with a temperature of ≥39 °C for >10 hours that is unresponsive to acetaminophen.

^c^Patients with early onset of CRS signs and symptoms (within 3 days of cell infusion), bulky disease, and comorbidities are at high risk of developing severe CRS.

^d^Simultaneous administration of corticosteroids and anti-IL-6 therapy or waiting to see if the patient responds to anti-IL-6 monotherapy before administering corticosteroids are both reasonable approaches (strategy used might vary depending on the CAR T cell products and/or risk factors).

CRS should be suspected if at least one of the following four symptoms or signs is present during the CRS-risk period after CAR T cell infusion: fever ≥38 °C; hypotension (defined as a systolic blood pressure (SBP) <(70 + (2 × age in years)) mmHg for patients aged 1–10 years or <90 mmHg for those aged >10 years, a change in SBP from baseline values, and/or a reduced requirement for chronic anti-hypertensive medications); hypoxia with an arterial oxygen saturation of <90% on room air; and/or evidence of organ toxicity as determined using the most recent Common Terminology Criteria for Adverse Events version 5.0 (CTCAE v5.0) grading system^99^ — bearing in mind specific considerations for paediatric patients^29,37,82^ (Tables 1,2). Frequent monitoring of complete blood count, coagulation, and chemistry profiles, including serum levels of liver enzymes, C-reactive protein, ferritin, and lactate dehydrogenase, might be useful for early detection of CRS.

While the criteria above provide general definitions of hypotension in children, it is important that the baseline blood pressure range of each child be defined before CAR T cell infusion so that relative hypotension from an elevated baseline is not missed. Reduced requirements for chronic anti-hypertensive medications can also indicate relative hypotension. Furthermore, because some symptoms can be caused by other concurrent conditions (for example, sinus tachycardia can have causes that range from crying to sepsis), care providers must use their clinical judgement to determine CRS attribution. Patients who develop fever with a temperature >38 °C, for example, should be assessed for infection using blood cultures and chest radiography; additional tests, such as viral PCR, respiratory viral screening, urine cultures, and CT of the chest, should be obtained as clinically indicated. Empiric antibiotic treatment should be initiated, and filgrastim should be considered if the patient is neutropenic and septic. Importantly, the patient’s orders should ensure triggering of such an escalation in care for a temperature >38 °C — some nursing units might be accustomed to higher temperature thresholds for intervention. This practice might require re-education of outpatient, emergency room, and triage staff that might not routinely care for patients treated with CAR T cells.

Careful vigilance for early recognition of haemodynamic shock in the child is crucial. Symptoms such as malaise, lethargy, weakness, oliguria, irritability, and reduced appetite are not always self-reported by younger children. Among infants, assessment of diapers will be critical to assess urine output and detect diarrhoea. We concur with recommendations that any patient requiring a rapid increase in the dose of vasopressors or exhibiting evidence of end-organ hypoperfusion should be treated intensively for grade 3 CRS, even if the vasopressor therapy required is ‘low dose’ according to the definition of Lee and colleagues^29,37^. For children with hypotension owing to CRS, an initial normal saline fluid bolus (10–20 ml/kg; maximum 1,000 ml) should be administered; if no improvement is observed, anti-IL-6 therapy should be initiated (Table 2). After administration of anti-IL-6 therapy, the decision to repeat additional fluid boluses versus starting vasopressors should involve consideration of the cardiac and fluid status of the child. For example, the administration of additional fluid boluses should be avoided in patients with underlying cardiac dysfunction and/or signs and symptoms of volume overload (such as pulmonary oedema). Additionally, early use of colloid solutions might be indicated because patients with CRS could potentially develop capillary leak and hypoalbuminaemia more rapidly than patients with sepsis, and the administration of additional fluid boluses might compromise pulmonary function by causing pulmonary oedema. Care should be taken not to trigger acute fluid overload, cardiogenic shock, and/or respiratory compromise. Consideration should be given to adrenal insufficiency in decisions of the initial choice of corticosteroid intervention, if needed (Table 2); patients with vasopressor-resistant hypotension attributed to adrenal insufficiency might respond to stress-dose hydrocortisone only and, thus, high doses of other lymphocytotoxic corticosteroids (dexamethasone or methylprednisolone) can be avoided. Transfer to an intensive-care unit should be considered early in this process (Table 1).

Grading criteria for CRS-related hypoxia have predominantly been based upon fraction of inspired oxygen (FiO_2_) requirements and the need for mechanical ventilation^29,37^. In recognition of the differences in acute respiratory distress syndrome (ARDS) between adult and paediatric patients, a 2015 publication by the Pediatric Acute Lung Injury Consensus Conference (PALICC) group provided paediatric-specific definitions for paediatric ARDS (P-ARDS)^100^; these criteria have since been applied among paediatric patients who have undergone HSCT^101,102^. We recommend application of the PALICC at-risk P-ARDS criteria for the CRS grading of hypoxia^100^ (Table 1). Accordingly, the grading of CRS-related hypoxia should be based on the use of high-flow oxygen and other non-invasive forms of mechanical ventilation, whereby flow rates and FiO_2_ are indicators of severity. For example, paediatric patients who require supplemental oxygen exceeding an FiO_2_ requirement of 40% or those who are receiving non-invasive mechanical ventilation should be classified as having grade 3 CRS and be managed accordingly (Table 2).

CTCAE v5.0 grading of organ toxicity provides an objective assessment tool, although high vigilance is important for prompt recognition of CRS among children. Sinus tachycardia (according to age-dependent definitions) is often the earliest sign of CRS^103^ (Table 1). Expected heart rate ranges should also incorporate the child’s baseline measurements at patient admission before CAR T cell infusion^104^. Acute kidney injury in children can be graded according to CTCAE v5.0 criteria using the Pediatric Risk, Injury, Failure, Loss, End-Stage Renal Disease (pRIFLE) and Kidney Disease: Improving Global Outcomes (KDIGO) definitions of oliguria and anuria^105,106^ (Tables 1,2).

In one study^20^, the use of high-dose steroids to treat CRS was associated with suppression of CAR T cell expansion and unfavourable patient outcomes. However, no adequately powered randomized studies investigating whether the administration of anti-IL-6 therapy and/or corticosteroids reduces the efficacy of CAR T cell therapy have been reported to date. Notably, the use of anti-IL-6 therapy and/or corticosteroids for the management of CRS (usually following CAR T cell expansion) has not negatively affected disease-free survival outcomes in larger cohorts^107,108^. Indeed, the anti-IL-6 receptor antibody tocilizumab is currently approved by the FDA for management of CRS^109^; patients weighing <30 kg are treated at a dose of 12 mg/kg, and those weighing ≥30 kg are treated at a dose of 8 mg/kg^110^ (Tables 1,2). Clinical responses following the administration of tocilizumab are often observed within 4 hours (ref.^33^). Patients who do not respond to the first dose of tocilizumab might be less likely to respond to subsequent repeat administration of this agent; for these patients, consideration should be given to administration of corticosteroids — weighing the risks and benefits of steroid use for CRS and the uncertain risk of suppressing CAR T cell expansion — and/or alternative agents, such as the anti-IL-6 monoclonal antibody siltuximab^37,38,111^. Corticosteroids can also be administered concurrently with repeat doses of tocilizumab (Table 2). When corticosteroids are used, the taper should be rapid and individualized according to the patient’s response. The initial choice of corticosteroid (hydrocortisone, dexamethasone, or methylprednisolone) will vary depending on institutional preference, protocol-specific guidelines, or product label specifications.

## CAR T cell therapy-related HLH

**Fig. 1:**
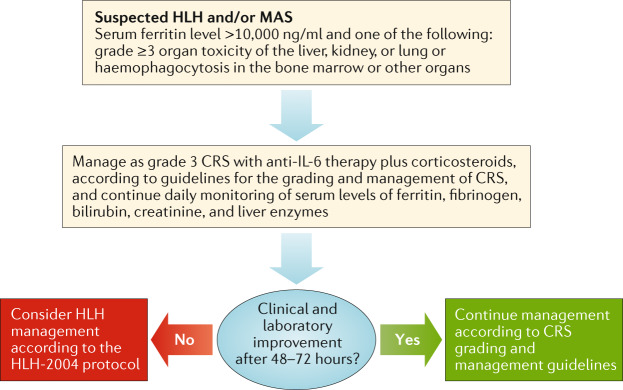
Proposed algorithm for the diagnosis and management of CAR T cell-related haemophagocytic lymphohistiocytosis and/or macrophage-activation syndrome. Chimeric antigen receptor (CAR) T cell-related haemophagocytic lymphohistiocytosis (HLH) and macrophage-activation syndrome (MAS) are serious, life-threatening complications of CAR T cell therapy and should be suspected when a patient has a serum ferritin level >10,000 ng/ml in association with grade ≥3 organ toxicities (liver, kidney, or lung) per Common Terminology Criteria for Adverse Events (version 5.0)^99^ and/or evidence of haemophagocytosis in the bone marrow or other organs. Patients should be managed as recommended for grade 3 cytokine-release syndrome (CRS) with close monitoring of inflammatory markers and organ function. If no clinical and laboratory improvement is observed after 48–72 hours, consider HLH management according to the HLH-2004 protocol^113^.

Haemophagocytic lymphohistiocytosis (HLH) is a rare syndrome with severe clinical sequelae that result from a dysregulated, hyperinflammatory immune response^112^ and can present in a primary (inherited) or a secondary form^113^. Secondary HLH is thought to occur in the context of an underlying immunological condition and, in the setting of autoimmune and inflammatory disorders, is often referred to as macrophage-activation syndrome (MAS). The diagnosis of HLH is made on the basis of the presence of mutations associated with primary HLH (such as mutations in *PRF1*, *UNC13D*, or *STX11*) and/or clinical and laboratory criteria, such as fever, cytopenias, hypertriglyceridaemia, hypofibrinogenaemia, elevated serum levels of ferritin and liver enzymes, haemophagocytosis, low or absent NK cell activity, and/or elevated soluble IL-2 receptor levels^113^. Differentiation of primary HLH from MAS can be difficult, and distinction of secondary CAR T cell-related HLH–MAS from CRS–CRES can be even more challenging owing to the overlapping symptoms associated with these conditions^16,29,37,112^. Future studies to define the role of genetic testing and functional analyses of NK cells might help identify patients who are at a disproportionately higher risk of this complication of CAR T cell therapy^30^. Nevertheless, diagnostic criteria for this rare toxicity have been provided in the guidelines for the management of adult patients treated with CAR T cell therapy published by Neelapu et al.^37^ in this journal. Children can also be diagnosed with CAR T cell-related HLH–MAS if they have a peak serum ferritin level >10,000 ng/ml during the CRS-risk period and develop any two of the following: grade ≥3 organ toxicities involving the liver, kidney, or lung; or haemophagocytosis in the bone marrow or other organs^37^. Patients who develop CAR T cell-related HLH–MAS can be treated simultaneously with anti-IL-6 therapy and corticosteroids (Fig. 1); responses to anti-IL-6 therapy alone might not be as common as in patients with CRS alone^33^. In addition, although HLH–MAS occurring after treatment with CAR T cells and other T cell-engaging therapies has typically been shown to resolve following administration of anti-IL-6 therapy and/or corticosteroids^30,62,96^, refractory cases can require additional therapy, including consideration of systemic and/or intrathecal therapy according to the HLH-2004 management guidelines^113^ or use of the IL-1 receptor antagonist anakinra^62,114^ (Table 1). Further research is needed, however, to optimize the diagnosis and treatment of CAR T cell-related HLH–MAS.

## CRES

Neurological symptoms associated with CAR T cell therapy, referred to as CRES, commonly present as a toxic encephalopathy with delirium, seizures, and/or cerebral oedema^29,37,62,82,115^. The earliest signs and symptoms of CRES can be subtle among children, and, thus, a paediatric skill set is required in order to determine the child’s baseline level of cognitive performance. Among adult patients, early presenting symptoms of CRES include inattention and impaired expression affecting language and handwriting^37^. Grading of CRES according to CTCAE v5.0 (ref.^99^) or previously published CRES algorithms for adult patients^29,37^ is not optimal among infants and younger children. The Cornell Assessment of Pediatric Delirium (CAPD)^116^ is a validated screening tool (Supplementary Table 2) for recognition of delirium among children and adolescents (from birth to 21 years old); the sensitivity and specificity of this tool are highest in patients aged <12 years. Use of CAPD with appropriate developmental anchor points^117^ enables developmentally appropriate delirium screening by nurses and other members of the health-care team at the bedside and is an important tool in the overall grading of CRES, as outlined in Table 3; a CAPD score >8 is indicative of delirium^116^. Alternatively, neurological assessment scoring, as previously described with the CARTOX 10-point assessment scale (CARTOX-10) grading system by Neelapu et al.^37^, can be used for patients aged ≥12 years who have cognitive abilities that are appropriate for these assessments (Table 3). We recommend that delirium screening with CAPD or other neurological assessments be performed at least twice per day among admitted patients and at least daily among outpatients during the high-risk period for CRES (the 4 weeks after CAR T cell infusion) (Table 1). The first neurological assessment from a nursing provider should occur at the end of their shift and be conducted concurrently with the incoming nurse. The severity of CRES can be labile; thus, assessments should be performed more frequently if a change from prior scores occurs and/or if a caregiver raises concerns. Indeed, the trend in CAPD scores within an individual patient is important; increasing scores can be used as a marker of CRES severity (Table 3).

**Table 3:** CAR T cell-related encephalopathy syndrome grading and management

Grade 1 CRES	Grade 2 CRES	Grade 3 CRES	Grade 4 CRES
Signs and symptoms			
For patients aged >12 years (with age-appropriate cognitive performance): • Grade 1 somnolence, confusion, encephalopathy, dysphasia, seizure (brief partial seizure without loss of consciousness), and/or tremor^a^ • Neurological assessment score 7–9 according to CARTOX-10 grading system^37^ For patients aged ≤12 years: • Grade 1 CNS toxicities as above and CAPD^116^ score <9	For patients aged >12 years (with age-appropriate cognitive performance): • Grade 2 somnolence, confusion, encephalopathy, dysphasia, seizure (brief generalized seizure), and/or tremor^a^ • Neurological assessment score 3–6 For patients aged ≤12 years: • Grade 2 CNS toxicities as above and CAPD score <9	For patients aged >12 years (with age-appropriate cognitive performance): • Grade 3 somnolence, confusion, encephalopathy, dysphasia, seizure (multiple seizures despite medical interventions), tremor and incontinence or motor weakness^a^, and/or elevated intracranial pressure (stage 1 or 2 papilloedema^b^ with CSF opening pressure <20 mmHg) • Neurological assessment score 0–2 For patients aged ≤12 years: • CAPD score ≥9	• Patient is critical, obtunded, and/or unable to perform CAPD • High-grade (stage 3–5) papilloedema^b^, CSF opening pressure ≥20 mmHg, or cerebral oedema • Life-threatening prolonged repetitive seizure • Requirement for invasive mechanical ventilation
Management			
• Vigilant supportive care with aspiration precautions and i.v. hydration • Withhold oral intake of food, medicines, and fluids and assess swallowing • Substitute all oral medications and/or nutrition with i.v. forms if swallowing is impaired • Avoid medications that cause CNS depression • Low doses of lorazepam (0.05 mg/kg (maximum 1 mg per dose) i.v. every 8 hours) or haloperidol (0.05 mg/kg (maximum 1 mg per dose) i.v. every 6 hours) can be used, with careful monitoring, for agitated patients • Neurology consultation • Fundoscopic exam to assess for papilloedema • MRI of the brain with and without contrast and diagnostic lumbar puncture with measurement of opening pressure; include MRI of the spine if focal peripheral neurological deficits have been observed. CT scan of brain can be performed if brain MRI is not feasible • Perform EEG: if no seizures on EEG, continue prophylactic treatment with levetiracetam (Box 1); if EEG shows non-convulsive status epilepticus, treat patient according to algorithm A (Box 4) • Consider anti-IL-6 therapy if CRES is associated with concurrent CRS	• Supportive care and neurological work-up as per grade 1 CRES • Administer anti-IL-6 therapy if associated with concurrent CRS • Dexamethasone 0.5 mg/kg (maximum 10 mg per dose) i.v. every 6 hours or methylprednisolone 1–2 mg/kg per day divided every 6–12 hours for CRES that is not associated with concurrent CRS or is refractory to prior anti-IL-6 therapy • Consider transfer to PICU if associated with grade ≥2 CRS (Table 2)	• Supportive care and neurological work-up as per grade 1 CRES • PICU transfer is recommended • Administer anti-IL-6 therapy if associated with concurrent CRS and if not administered previously • Dexamethasone 0.5 mg/kg (maximum 10 mg per dose) i.v. every 6 hours; increase to 20 mg i.v. every 6 hours if patient is refractory to initial doses or methylprednisolone 1–2 mg/kg per day divided every 6–12 hours around the clock if symptoms worsen despite anti-IL-6 therapy or for CRES without concurrent CRS • Continue corticosteroid treatment until improvement to grade 1, and then taper or stop • For patients with stage 1 or 2 papilloedema^b^ with a CSF opening pressure <20 mmHg, treat according to algorithm A (Box 5) • Consider repeat neuro-imaging (CT or MRI) every 2–3 days if ≥3 grade CRES persists	• Supportive care and neurological work-up as per grade 1 CRES • PICU monitoring; consider mechanical ventilation for airway protection • Neurosurgical evaluation • Consider repeating CT scans • Obtain chemistry panels frequently (every 6–8 hours), adjust medication and provide osmotherapy to prevent rebound cerebral oedema, renal failure, hypovolemia and/or hypotension, and electrolyte abnormalities • Anti-IL-6 therapy and repeat neuro-imaging as for grade 3 CRES • Consider high-dose corticosteroids (for example, methylprednisolone 1 g per day i.v. for 3 days followed by rapid taper) • Continue corticosteroids until improvement to grade 1 CRES, and then taper • For patients with convulsive status epilepticus, treat according to algorithm B (Box 4) • For patients with stage 3, 4, or 5 papilloedema, CSF opening pressure ≥20 mmHg, or cerebral oedema, treat per algorithm B (Box 5)

Early recognition of and intervention for chimeric antigen receptor (CAR) T cell-related encephalopathy syndrome (CRES) are essential to avoid life-threatening complications. CRES should be suspected if any of the above listed signs and symptoms are present within the first 4 weeks of CAR T cell therapy. CRES grading including patient history, physical examination, and CAR T Cell Therapy-Associated Toxicity 10-point assessment scale (CARTOX-10) neurological assessment score^37^ or the Cornell Assessment of Pediatric Delirium (CAPD) tool^116^ should be performed at least twice a day and when a change in the patient's clinical status is observed. The trend in CAPD scores within an individual patient is important; increasing scores can be used as a marker for CRES severity. CNS, central nervous system; CRS, cytokine-release syndrome; CSF, cerebrospinal fluid; EEG, electroencephalography; FiO_2_, fraction of inspired oxygen; i.v., intravenous; PICU, paediatric intensive-care unit.

^a^Graded according to the Common Terminology Criteria for Adverse Events (CTCAE) version 5.0 guidelines^99^; CTCAE-defined neurological toxicities should be assessed for aetiology, in a similar manner to fevers, and if the toxicities are thought to be attributable to CRES, then symptoms should be treated according to the management recommendations provided.

^b^Papilloedema scoring according to the modified Frisen Scale^147^.

The onset of CRES can be biphasic, occurring concurrently with CRS and/or after CRS has resolved, and the precise pathophysiology remains unclear — although evidence implicates a combination of endothelial activation in the CNS, elevated cytokine levels in the cerebrospinal fluid (CSF), and cerebral T cell infiltration^118,119^. The use of anti-IL-6 therapy seems to be more effective for the management of CRES that occurs concurrently with CRS. Patients who develop CRES can also benefit from early corticosteroid administration^29,37,38^ (Table 3). CRES is generally reversible; however, cerebral oedema and death have been reported^37,64,120–122^. No adequately powered randomized studies to identify patients who are at disproportionately higher risk of CRES have been reported to date. We recommend that recipients of CAR T cell therapy with CNS disease or a history of seizures receive anti-seizure prophylaxis with levetiracetam (10 mg/kg, up to a maximum of 500 mg per dose) every 12 hours for 30 days following infusion (or through the CRES-risk period as described during pivotal studies and with subsequent emerging data)^9,27,37^ (Box 1). Levetiracetam is generally well-tolerated, with a minimal risk of adverse drug interactions, although dose adjustments might be necessary in the setting of renal dysfunction and are not thought to affect cytokine levels^37,123,124^. Neurology consultation should be considered if the patient develops grade 1 CRES (Table 3) and/or for specialized screening for papilloedema. Patients should be closely monitored for signs and symptoms of cerebral oedema. Status epilepticus can be managed according to institutional guidelines (our recommended management approaches are provided in Box 4). In general, first-line anti-seizure medications with unfavourable cardiotoxicity profiles (such as lacosamide and phenytoin) should be avoided when possible. Increased intracranial pressure (CSF opening pressure ≥20 mmHg or clinical signs of increased intracranial pressure) will require intensive-care management and osmotherapy (a management algorithm that can be tailored according to institutional guidelines is proposed in Box 5). A neurosurgery consultation should be considered, and brain scans can help guide patient management. Routine chemistry panels should be monitored more frequently (every 6–8 hours) and medications adjusted accordingly to prevent rebound cerebral oedema, renal failure, hypovolemia and/or hypotension, and electrolyte abnormalities. General grading and management guidelines for CRES are outlined in Table 3 (these can be tailored according to product-specific approved label instructions and/or study protocols). CRES can occur as a later complication and, in some patients, after discharge from hospital; therefore, caregivers and/or the patient should be given appropriate anticipatory guidance and appropriate education before being discharged from hospital. Moreover, patients should have a caregiver available who can observe for signs of CRES and seek prompt intervention for at least 4 weeks (or through the CRES-risk period) after CAR T cell infusion.

Box 4 Proposed management algorithms for status epilepticus **Algorithm A: non-convulsive status epilepticus**

- Assess circulation, airway, and breathing (CAB) and provide airway protection interventions, provide high-flow O_2_, and check blood glucose level
- Lorazepam 0.05 mg/kg (maximum 1 mg) intravenous (i.v.); repeat dose every 5 mins (to a maximum of 4 doses) to control electrographical seizures
- Levetiracetam 40 mg/kg (maximum 2,500 mg) i.v. bolus (in addition to maintenance dose)
- If seizures persist, transfer patient to paediatric intensive-care unit (PICU) and add phenobarbital i.v. at a loading dose of 10–20 mg/kg (maximum 1,000 mg)
- Administer corticosteroids (see Table 3)
- Maintenance doses of anticonvulsant drugs after resolution of status epilepticus are as follows:

- Lorazepam 0.05 mg/kg (maximum 1 mg) i.v. every 8 hours for 3 doses
- Levetiracetam 15 mg/kg (maximum 1,500 mg) i.v. every 12 hours
- Phenobarbital 1–3 mg/kg i.v. every 12 hours

**Algorithm B: convulsive status epilepticus**

- Assess CAB and provide airway protection interventions, administer high-flow O_2_, and check blood glucose level
- Transfer patient to PICU
- Lorazepam 0.1 mg/kg (maximum 2 mg) i.v.; repeat dose after at least 1 minute (to a maximum of 2 doses) to control seizures
- Levetiracetam 40 mg/kg (maximum 2,500 mg) i.v. bolus (in addition to maintenance dose)
- If seizures persist, add phenobarbital i.v. at a loading dose of 10–20 mg/kg (maximum 1,000 mg)
- Administer corticosteroids (see Table 3)
- Maintenance doses after resolution of status epilepticus are as follows:

- Lorazepam 0.05 mg/kg (maximum 1 mg) i.v. every 8 hours for 3 doses
- Levetiracetam 30 mg/kg i.v. every 12 hours or increase the prophylaxis dose by 10 mg/kg (to 20 mg/kg) i.v. every 12 hours (maximum dose of 1,500 mg)
- Phenobarbital 1–3 mg/kg i.v. every 12 hours

- Continuous electroencephalography monitoring if seizures are refractory

Box 5 Proposed management algorithms for increased intracranial pressure **Algorithm A: stage 1–2 papilloedema with CSF opening pressure <20 mmHg and without evidence of cerebral oedema**

Acetazolamide 15 mg/kg (maximum 1,000 mg) intravenous (i.v.) followed by 8–12 mg/kg (maximum 1,000 mg) i.v. every 12 hours; monitor renal function and acid–base balance once or twice daily and adjust dose accordingly

**Algorithm B: management of stage 3–5 papilloedema, any evidence of cerebral oedema on imaging studies, or CSF opening pressure ≥20 mmHg**

Use high-dose corticosteroids according to recommendations for grade 4 chimeric antigen receptor (CAR) T cell-related encephalopathy syndrome (CRES; see Table 3) along with the following measures for the management of cerebral oedema:

- Elevate head of bed to an angle of 30 degrees
- Hyperventilation to achieve target PaCO_2_ of 30–40 mmHg during the acute management of intracranial hypertension (or acute management of intracranial hypertension according to accepted institutional guidelines)
- Hyperosmolar therapy with either 20% mannitol or hypertonic saline (3%)

- Mannitol: initial dose of 0.5–1 g/kg; maintenance dose 0.25–1 g/kg every 6 hours (check metabolic profile and serum osmolality every 6 hours, and hold mannitol if serum osmolality is ≥320 mOsm/kg or osmolality gap is ≥40)
- Hypertonic 3% saline: initial dose 5 ml/kg i.v. over 15 mins; maintenance dose 1 ml/kg per hour i.v. to reach a target serum sodium level of 150–155 mEq/l (check electrolytes every 4 hours, and hold infusion if sodium level is >155 mEq/l)

- If patient has an Ommaya reservoir, drain CSF to a target opening pressure of <20 mmHg
- Consider neurosurgery consultation and i.v. anaesthetics for burst-suppression pattern on electroencephalography
- Perform metabolic profiling every 6 hours, daily CT of the head, and adjust above medications to prevent rebound cerebral oedema, renal failure, electrolyte abnormalities, hypovolemia, and hypotension

CSF, cerebrospinal fluid; mEq, milliequivalents; mOsm, milliosmole; PaCO_2_: partial pressure of carbon dioxide in arterial blood.

## Long-term follow-up assessment

Careful long-term follow-up assessment of patients receiving CAR T cell therapy is important. Management of on-target, off-tumour effects should be well coordinated between treatment and referring centres if the patient returns to local providers following therapy.

For example, B cell aplasia and hypogammaglobulinaemia or agammaglobulinaemia are commonly seen in patients treated with anti-CD19 CAR T cells. These adverse effects require long-term replacement with intravenous immunoglobulins (IVIGs). We recommend intervention to maintain serum immunoglobulin levels above 400 µg/l with IVIGs as well as consideration of IVIG administration to provide specific immunity during active infection, irrespective of immunoglobulin levels^63,125^. B cell aplasia has been associated with progressive multifocal leukoencephalopathy (PML)^126,127^; thus, patients should be closely monitored for neurological signs and symptoms that are suggestive of PML (neuropsychological deficits, progressive dementia, apraxia, or visual and motor deficits)^126^ until the resolution of B cell aplasia.

Data from studies of the effectiveness and safety of immunization with inactive or live vaccines in patients treated with adoptive T cell therapies have not been reported to date. We recommend careful assessment of immune reconstitution after lymphodepletion and CAR T cell infusion; the findings should guide decisions regarding antimicrobial prophylaxis and re-vaccination^63,128^.

Patients treated with CAR T cell therapy are at risk of disease relapse and/or the development of secondary neoplasms. In addition, the use of a replication-competent viral vector during CAR manufacturing could pose a theoretical risk to patients and/or their close contacts^129^. Long-term clinical monitoring is important for detection of these complications and is mandated by the FDA for all such gene therapies^130,131^.

Local and national registries to capture the outcomes, acute complications, and late effects of CAR T cell therapies might enable the establishment of quality benchmarks, facilitate retrospective research, recognize potential delayed toxicities, and ultimately improve future care. As the Center for International Blood and Marrow Transplant Research (CIBMTR) develops a registry for patients receiving CAR T cell therapy, we recommend that consideration be given to the reporting of variables that are directly retrievable from electronic medical records to ensure accuracy and minimize the infrastructural burden required for comprehensive reporting. Given that toxicity grading systems are likely to evolve over time, entry of primary variables seems more useful in the long term. Furthermore, many of these patients require intensive-care support, and therefore prospective collaborations with intensive-care registries, such as Virtual paediatric intensive-care unit (PICU) Systems (VPS)^132^, should be considered (Table 1). This approach could enable accurate data entry of cell-therapy variables into the CIBMTR registry by cell-therapy programmes, with concurrent entry of data on intensive-care variables into an appropriate registry by paediatric critical care teams.

## CAR T cell therapy as a bridge to HSCT

In ELIANA^8^, the largest study of CAR T cell therapy involving paediatric patients performed to date, 83% of the infused patients (*n* = 63) achieved minimal residual disease (MRD)-negative complete remission (CR) or CR with incomplete haematological recovery (CRi). After a median follow-up duration of 4.8 months from response, the median CR and/or CRi duration was not reached (range 1.2 months to >14.1 months). The results of prediction-based modelling suggest that more than half of the patients who received tisagenlecleucel on the ELIANA trial will be alive at 5 years after treatment^133^. The actual allogeneic HSCT (allo-HSCT) rate among those who achieved a CR or CRi was 12% in the ELIANA trial^8^.

In another paediatric study^46^, CD4^+^ and CD8^+^ T cells transfected with an anti-CD19 CAR construct containing a 4-1BB co-stimulatory domain using a lentiviral vector were administered to 45 children and young adults with pre-B cell ALL; 93% of the patients achieved MRD-negative remission by day 21. However, the estimated 12-month event-free survival was 50.8%, with the majority of these patients unfortunately experiencing disease relapse^46^. The persistence of functional anti-CD19 CAR T cells was assessed by measuring the duration of B cell aplasia using flow cytometry; the median duration of B cell aplasia was 3 months (95% CI 2.07–6.44)^46^. In this study^46^, 11 of 40 (28%) patients who were in CR underwent allo-HSCT, and 2 of these 11 patients subsequently experienced CD19^+^ leukaemia relapse.

In an open-label, phase I, dose-escalation study of anti-CD19 CAR T cells (containing a CD28 co-stimulatory domain and manufactured using a retroviral vector) involving children and young adults with ALL or non-Hodgkin lymphoma performed by the US NIH, the CR rate was 66.7%. Following remission, 10 of 12 (83%) patients who achieved MRD-negative remission underwent HSCT and remained disease-free at the time of publication of the data^22^.

At this time, whether CAR T cell therapy is a definitive treatment remains unclear. While strategies to understand antigen-escape mechanisms and to increase rates of long-term remission are developed^134^, allo-HSCT can reasonably be considered for patients with haematological malignancies who have achieved remission following CAR T cell therapy. Alternatively, as CAR T cell product-specific data matures, it might also be reasonable to consider CAR T cell therapy as a definitive treatment. The decision to proceed with allo-HSCT should be based upon the candidate meeting standard eligibility requirements, and the long-term outcomes associated with the specific CAR T cell product used should be considered in the risk–benefit assessment.

## Ethical considerations

Currently, CAR T cell therapy for paediatric patients is available for only those with high-grade, relapsed and/or refractory ALL. Remission rates among children with relapsed and/or refractory ALL, who previously had no curative options, have been impressive with current CAR T cell therapies^97^. Nevertheless, not all children with relapsed and/or refractory ALL are appropriate candidates for this therapy. Patients who do not have a reasonable expectation of survival between leukapheresis and CAR T cell administration or whose survival after CAR T cell therapy is expected to be limited by other comorbidities should not be considered as candidates for this treatment. Among these groups, the risks of primary disease progression must be weighed against the risk of accelerating mortality and/or causing severe disability that could potentially be associated with CAR T cell therapy^135^.

## Financial and health-system considerations

We understand that value in health care is determined by patient outcomes balanced against costs. The current estimated cost of standard of care CAR T cell therapy for children with ALL is high^136,137^. Moreover, the ancillary administrative and supportive care service (including management of complications, intensive-care unit stays, and frequent hospitalization) costs can be substantially higher than the CAR T cell product price tag^138^. We strongly encourage consideration of the quality-adjusted life years gained for paediatric patients who can potentially achieve long-term remission as a result of this therapy and encourage all efforts to reduce the costs of care^137,139,140^ (Table 1). We anticipate that advances in CAR T cell technology will improve our understanding of the pathophysiology of CRS–CRES and facilitate the discovery of predictive biomarkers with which to identify patients requiring early intervention with available supportive care, which will subsequently lead to improved outcomes and reductions in the cost of this care, in addition to biomarkers for identifying in advance patients who are unlikely to respond. As payers and health systems determine coverage benefits, it is important that the specific needs of all children be considered and that access is granted to CAR T cell therapy and the associated supportive care (encompassing baseline assessments, inpatient observation, and essential supportive care when necessary).

Health institutions are encouraged to provide access to CAR T cell therapies; however, adequate strategic and operational planning and preparation are needed to ensure the safe delivery of such treatments. We recommend that programmes seek immune effector cell (IEC) accreditation by the FACT as a voluntary means of ensuring adherence to quality standards^55^ (Table 1). FACT accreditation will require an established quality-assurance programme at the institution, as well as education and ongoing training for clinical staff. Institutions that offer CAR T cell therapy should support rigorous quality assurance, data management, clinical services, and education programmes for interdisciplinary staff involved in the care of patients who receive this treatment. Additionally, emergency medical services, community hospitals, and local triage facilities will require high vigilance to recognize and promptly escalate care in the event that a patient treated with CAR T cell therapy presents to their facility in an emergency.

## Nursing considerations

The availability of skilled interdisciplinary staff, including nurses, is an essential requirement for safe administration of CAR T cell therapy to paediatric patients. Owing to the need for close medical attention among these patients, communication between coordinators, medical care providers, and nursing administration is important to ensuring that CAR T cell infusions are considered together with staffing decisions^141,142^. Inpatient and outpatient nursing units, triage facilities, emergency-care departments, and intensive-care units all need available nursing staff that have completed all required competencies to care for patients treated with CAR T cells. These nurses can assist in the rapid recognition of CRS–CRES and help avoid iatrogenic errors (for example, administration of steroids as a routine pre-medication). Visual cues, such as flags in the patient chart or patient bracelets (similar to ‘fall risk’ identifiers), can help care teams to quickly recognize recipients of CAR T cells, even during electronic medical record ‘downtime procedures’. Discharge protocols should ensure comprehensive education of the caregiver and patient about signs and symptoms of CRS and CRES, and the patient should also be given a wallet or REMS identification card for their specific CAR T cell product. Most importantly, recipients of CAR T cells should be instructed to immediately alert all providers that they have received this therapy, especially if presenting to a facility outside of their original treatment centre.

## Pharmacy considerations

As CAR T cell therapies transition from experimental therapies to standard-of-care treatments, institutional pharmacists must be engaged in the development of policies and protocols to optimize supportive care. Additionally, REMS programmes are likely to accompany new therapy approvals by the FDA. For instance, the REMS programme for tisagenlecleucel requires institutions to have a minimum of two doses of tocilizumab available on site for each patient at risk of CRS–CRES in order to enable immediate administration^143^. New CAR T cell therapies might also require an adequate stock of other agents directed at ameliorating CRS and/or CRES and other supportive therapies (such as rituximab and cetuximab for the aforementioned products with integrated suicide or safety switches)^49,56,61^. Institutional pharmacies will need to ensure adequate training of pharmacy staff regarding the recognition and management of CRS and CRES and the development of policies and protocols to ensure adequate stock and the prompt dispensation of supportive therapies^55^.

## Emergency contingency planning

Institutions involved in the administration of CAR T cell therapies are encouraged to develop programme-specific emergency plans that take into account the particular needs of patients exposed to these treatments^144^. Many patients will be required to live within a specified distance of the treating hospital for a predefined amount of time after CAR T cell infusion. Should acute toxicities develop, this stipulation will ensure prompt access to care, which will help to mitigate against additional complications. When possible, institutions should develop protocols to overcome barriers to care for these patients in the event of natural disasters and/or disruption of services. Prespecified electronic medical record downtime procedures, evacuation plans that include access to anti-IL-6 therapies, and the consideration of admission of outpatients at risk of CRS–CRES before an anticipated natural disaster are examples of disaster planning protocols^145,146^.

## Conclusions

To achieve improvements in CAR T cell therapy and the requisite supportive care, and thereby sustain CR rates among children, we recommend that enrolment of children in trials of novel agents be facilitated at the earliest acceptable time points. In this regard, application of CAR T cell therapies for treatment of solid tumours and other haematological malignancies in children is being explored (NCT03126864, NCT01953900, NCT02311621, and NCT03056339). Moreover, consideration of earlier or upfront use of CAR T cell therapy might spare patients the acute and long-term toxicities associated with traditional chemotherapy and/or radiation regimens. Whether CAR T cell therapy should be followed by allo-HSCT or repeat CAR T cell infusions also needs to be explored. Future studies aimed at improving the persistence of CAR T cells and thereby inducing long-term remission without the need for further therapy are an important requirement. An overarching commitment to improve patient outcomes, especially among children with limited or no therapeutic options, is key.

Currently, the time required from leukapheresis to manufacture CAR-expressing cells is a major limitation of CAR T cell therapy. Efforts to explore third-party, off-the-shelf allogeneic approaches to therapy with CAR T cells or other immune effectors, such as CAR NK cells, are exciting potential alternatives^60^.

Additional long-term prospective studies to understand the pathophysiology and develop early recognition and optimal treatment strategies for CRS and/or CRES are needed. Such studies should involve broad collaborative efforts because these toxicities are multifaceted and necessitate interdisciplinary care. Response and adverse event rates, as well as late effects, are likely to vary depending on various host, disease, and CAR T cell characteristics; therefore, a comprehensive and robust registry is needed to guide future efforts to optimize and expand the use of CAR T cell therapy.

## Supplementary Information

Supplementary Tables
