# Supplementary material for: Management guidelines for paediatric patients receiving chimeric antigen receptor T cell therapy
Source: Nat Rev Clin Oncol. 2018 Aug 6;16(1):45–63. doi: 10.1038/s41571-018-0075-2 (PMC7096894; doi:10.1038/s41571-018-0075-2)
Supplement: Supplementary file 1 — Supplementary Tables [file 41571_2018_75_MOESM1_ESM.pdf]

nature REVIEWS CLINICAL ONCOLOGY

SUPPLEMENTARY INFORMATION

In format as provided by the authors

# Management guidelines for paediatric patients receiving chimeric antigen receptor T cell therapy

*Kris M. Mahadeo Sajad J. Khazal, Hisham Abdel-Azim, Julie C. Fitzgerald, Agne Taraseviciute, Catherine M. Bollard, Priti Tewari, Christine Duncan, Chani Traube, David McCall, Marie E. Steiner, Ira M. Cheifetz, Leslie E. Lehmann, Rodrigo Mejia, John M. Slopis, Rajinder Bajwa, Partow Kebriaei, Paul L. Martin, Jerelyn Moffet, Jennifer McArthur, Demetrios Petropoulos, Joan O'Hanlon Curry, Sarah Featherston, Jessica Foglesong, Basirat Shoberu, Alison Gulbis, Maria E. Mireles, Lisa Hafemeister, Cathy Nguyen, Neena Kapoor, Katayoun Rezvani, Sattva S. Neelapu and Elizabeth J. Shpall, the Pediatric Acute Lung Injury and Sepsis Investigators (PALISI) Network*

https://doi.org/10.1038/s41571-018-0075-2



© 2018 Macmillan Publishers Limited, part of Springer Nature. All rights reserved.

Supplementary Table 1 | **Levels of evidence for clinical practice guidelines**

**a | Definitions of levels of evidence**

| Level of evidence | Definition |
|---|---|
| **Ia** | Evidence from a meta-analysis of randomized controlled trials |
| **Ib** | Evidence from at least one randomized controlled trial |
| **IIa** | Evidence from at least one controlled study without randomization |
| **IIb** | Evidence from at least one other type of quasi-experimental study |
| **III** | Evidence from non-experimental descriptive studies, such as comparative studies, correlation studies, and case–control studies |
| **IV** | Evidence from expert committee reports or opinions or clinical experience of respected authorities, or both |

**b | Definitions of grades of evidence and their implications for clinical practice recommendations**

| Grade | Descriptor | Level of Evidence | Implications for clinical practice |
|---|---|---|---|
| **A** | **Strong recommendation** | Directly based on category I evidence | Clinicians should follow a strong recommendation unless a clear and compelling rationale for an alternative approach is present |
| **B** | **Recommendation** | Directly based on category II evidence, or extrapolated recommendation from category I evidence | Generally, clinicians should follow a recommendation but should remain alert to new information |
| **C** | **Optional** | Directly based on category III evidence, or extrapolated recommendation from category I or II evidence | Clinicians should be flexible in their decision-making regarding appropriate practice, although they may set bounds on alternatives |
| **D** | **Optional** | Directly based on category IV evidence, or extrapolated recommendation from category I, II, or III evidence | Clinicians should consider all options in their decision-making and be alert to new published evidence that clarifies the balance of benefit versus harm |

Table adapted with permission from Ref.[S1], BMJ.

Supplementary Table 2 | **Cornell Assessment of Pediatric Delirium[S3] tool with developmental anchor points[S4]**

Please answer the following based on your interactions with the patient over the course of your shift:

| | Never 4 | Rarely 3 | Sometimes 2 | Often 1 | Always 0 |
|---|---|---|---|---|---|
| 1. Does the child make eye contact with the caregiver? | | | | | |
| 2. Are the child's actions purposeful? | | | | | |
| 3. Is the child aware of his/her surroundings? | | | | | |
| 4. Does the child communicate needs and wants? | | | | | |
| | Never 0 | Rarely 1 | Sometimes 2 | Often 3 | Always 4 |
| 5. Is the child restless? | | | | | |
| 6. Is the child inconsolable? | | | | | |
| 7. Is the child underactive-very little movement while awake? | | | | | |
| 8. Does it take the child a long time to respond to interactions? | | | | | |

For patients aged 1–2 years, the following serve as guidelines to the corresponding questions (1–8):

1. Holds gaze. Prefers primary parent. Looks at speaker.
2. Reaches and manipulates objects, tries to change position, if mobile may try to get up
3. Prefers primary parent, upset when separated from preferred caregivers. Comforted by familiar objects (for example, a blanket or stuffed animal)
4. Uses single words or signs
5. No sustained calm state
6. Not soothed by usual comforting actions, for example, singing, holding, talking, and reading
7. Little if any play, efforts to sit up, pull up, and if mobile crawl or walk around
8. Not following simple directions. If verbal, not engaging in simple dialogue with words or jargon

Table adapted with permission from Ref.[S4], CUP.

**Supplementary references**


S1. Shekelle, P. G., Woolf, S. H., Eccles, M. & Grimshaw, J. Developing clinical guidelines. *West J. Med.* **170**, 348–351 (1999).

S2. Kalliainen, L. K; ASPS Health Policy Committee. ASPS clinical practice guideline summary on reduction mammaplasty. *Plast. Reconstr. Surg.* **130**, 785–789 (2012).

S3. Traube, C. et al. Cornell Assessment of Pediatric Delirium: a valid, rapid, observational tool for screening delirium in the PICU. *Crit. Care Med.* **42**, 656–663 (2014).

S4. Silver, G., Kearney, J., Traube, C. & Hertzig, M. Delirium screening anchored in child development: the Cornell Assessment for Pediatric Delirium. *Palliat. Support. Care* **13**, 1005–1011 (2015).
